# Nutrient Digestibility, Microbial Fermentation, and Response in Bacterial Composition to Methionine Dipeptide: An In Vitro Study

**DOI:** 10.3390/biology11010093

**Published:** 2022-01-07

**Authors:** Fanlin Kong, Yanfang Liu, Shuo Wang, Yijia Zhang, Wei Wang, Hongjian Yang, Na Lu, Shengli Li

**Affiliations:** 1 The State Key Laboratory of Animal Nutrition, Beijing Engineering Technology Research Center of Raw Milk Quality and Safety Control, College of Animal Science and Technology, China Agricultural University, Beijing 100193, China; fanlinkong1126@163.com (F.K.); b20213040351@cau.edu.cn (S.W.); wei.wang@cau.edu.cn (W.W.); yang_hongjian@cau.edu.cn (H.Y.); 2 Beijing Jingwa Agricultural Science & Technology Innovation Center, Beijing 100193, China; 13611235024@163.com; 3 Laboratory of Anatomy of Domestic Animals, College of Veterinary Medicine, China Agricultural University, Beijing 100193, China; yijiazhang202111@163.com

**Keywords:** methionine dipeptide, methionine, urea, in vitro, nutrient digestibility, bacterial composition

## Abstract

**Simple Summary:**

The rumen microbiota plays an important role in maintaining microbiota homeostasis and promoting milk production synthesis through utilizing amino acids and non-protein nitrogen. Furthermore, various nitrogen sources have shown distinct effects on microbial growth rates. The methionine dipeptide (MD) is a bioactive peptide consisting of two methionine (Met) residues linked by a peptide bond. Although the role of MD in milk protein synthesis is established, little is known about its role in bacterial fermentation. The present study demonstrates that the various nitrogen sources could reshape microbiota differently, and MD could be more efficient than free Met in the rumen to support acetate producer growth. Our study provides some new insights into the relationship between ruminal microbiota of dairy cows and small peptides and points to potential strategies to effectively enhance the health condition and digestion ability of dairy cows.

**Abstract:**

It is well known that the methionine dipeptide (MD) could enhance the dairy cows milking performance. However, there is still a knowledge gap of the effects of MD on the rumen fermentation characteristics, microbiota composition, and digestibility. This experiment was conducted to determine the effect of different nitrogen sources with a total mixed ration on in vitro nutrient digestibility, fermentation characteristics, and bacterial composition. The treatments included 5 mg urea (UR), 25.08 mg methionine (Met), 23.57 mg MD, and no additive (CON) in fermentation culture medium composed of buffer solution, filtrated Holstein dairy cow rumen fluid, and substrate (1 g total mixed ration). Nutrient digestibility was measured after 24 h and 48 h fermentation, and fermentation parameters and microbial composition were measured after 48 h fermentation. Digestibility of dry matter, crude protein, neutral detergent fiber (NDF), and acid detergent fiber (ADF) in the MD group at 48 h were significantly higher than in the CON and UR groups. The total volatile fatty acid concentration was higher in the MD group than in the other groups. In addition, 16S rRNA microbial sequencing results showed MD significantly improved the relative abundances of *Succinivibrio*, *Anaerotruncus*, and *Treponema_2*, whereas there was no significant difference between Met and UR groups. Spearman’s correlation analysis showed the relative abundance of *Succinivibrio* and *Anaerotruncus* were positively correlated with gas production, NDF digestibility, ADF digestibility, and acetate, propionate, butyrate, and total volatile fatty acid concentrations. Overall, our results suggested that the microbiota in the fermentation system could be affected by additional nitrogen supplementation and MD could effectively enhance the nutrient utilization in dairy cows.

## 1. Introduction

Peptides, which are generally derived from diet, play a vital role in animal physiology by acting as hormones, neurotransmitters, growth factors, and protein synthesis (body protein, milk protein, etc.) [1]. Consequently, peptides are becoming more popular in nutrition and pharmaceutical research [1,2]. Methionine dipeptide (MD) is a bioactive peptide consisting of two methionine (Met) residues linked by a peptide bond. MD, a crucial nutrient for milk protein gene expression, was found to be more effective than free Met in recent in vitro experiments [3,4]. As a result of the oligopeptide transporter 1 in the jejunum and ileum of dairy cows, MD has been proposed as a feed additive with the potential to enhance milk protein synthesis [5].

Ruminants utilize a wide range of dietary substrates that are indigestible for monogastric animals via rumen microbial fermentation. As a result, the rumen microbial fermentation is crucial for the growth and production of ruminants [6,7,8], and evaluating the effects of MD on rumen fermentation is essential for its application. Some studies have shown that small peptide is initially hydrolyzed into a free amino acid in the gastrointestinal tract [9,10], while other studies found that small peptides could be absorbed in intact peptide [11,12]. Hence, MD may provide microorganisms with Met and MD together. For dairy cows, Met is the most limiting amino acid [2]. Met supplementation in a rumen-protected form can improve milk fat [13], milk protein [14], and milk production in dairy cows [15]. In a recent study, soybean oil-induced milk fat depression was associated with significant alterations in the ruminal microbiota, which could be mitigated by rumen-protected Met [13]. Furthermore, Met expanded the proportion of cellulolytic bacteria (*Ruminococcus albus*) and increased microbial protein, and volatile fatty acid synthesis in an in vivo study [16]. Hence, Met could interact with bacteria and ultimately support better fermentation in the rumen [13,16].

Peptides have been found to improve microbial protein yield and rumen fermentation ability [17,18,19]. Rather than amino acids or ammonia, rumen bacteria can utilize nitrogen from peptides [20,21,22]. Fu et al. [18] demonstrated 1.8 mM peptide maximized microbial efficiency when ammonia nitrogen was not limiting. Meanwhile, non-structural carbohydrate-fermenting bacteria required 67% of their nitrogen from peptides or amino acids, and the efficiency of microbial uptake of peptide-nitrogen was 80%, according to Russel et al. [23,24]. In addition, Brooks et al. [25] reported that rumen degradable peptide supply improved microbial efficiency. Although these studies have indicated the significance of peptides, investigations using the 16S rRNA sequencing technique on microbiota composition are limited. Unlike free Met, most of the functional studies of MD have focused on the underlying mechanisms of MD’s effects on milk protein synthesis in bovine mammary epithelial [4,11,12], with fewer findings of the effects of MD on rumen fermentation. Therefore, it is essential to evaluate the effects of MD on rumen fermentation and bacterial composition, as well as to see if MD can be used as a feed additive to improve dietary digestibility in the rumen.

In this study, we aimed to determine the effects of supplementing MD, Met, and urea, in a total mixed ration (TMR), individually, on fermentation characteristics and bacterial composition in vitro, and to consider the possibility of MD being a preferred additive over Met. The incubation of substrate with rumen fluid in an In vitro automated trace gas recording system has been frequently utilized to assess the effect of a feed additive on rumen fermentation and digestibility [26,27]. Although in vitro simulation rumen system results cannot completely replace or reflect those obtained from in vivo studies, they do provide a rapid and less expensive alternative instead of in vivo feeding studies [27,28]. We hypothesized that the effects of MD on rumen fermentation and microbiota could be different from those of Met and that it may be helpful to dietary nutrient degradation.

## 2. Materials and Methods

### 2.1. Animals

The donor animals and experimental procedures were approved by the Institutional Animal Care and Use Committee of China Agricultural University (approval number: CAU2021009-2). We conducted this experiment in May. Three rumen-cannulated lactating Holstein dairy cows (36.4 ± 2.04 kg/d milk yield, 131 ± 18 days in milk) from China ZhongDi Dairy Holdings Company Limited (40°11′ N 116°88′ E, Beijing, China) were used as the donors of rumen fluid. The cows were fed thrice daily at 07:30, 13:30, and 18:30, and milked thrice daily at 09:00, 14:00, and 19:00. The ingredients and nutrient composition of the TMR are listed in Table 1. After the morning feeding, approximately 3 L of rumen fluid was collected from each cow at 09:30 h. In detail, we took ruminal content through the cannula and put it into a steel artificial squeezer. Then, the rumen fluid was juiced and filtered through four layers of cheesecloth, transferred to a prewarmed thermos bottle, then combined and cultivated in the laboratory with CO_2_ at 39 ℃ for subsequent in vitro experiment. The pH of the rumen fluid was measured right after juicing by using a portable pH meter (S2-Meter, Mettler Toledo International Co., Ltd., Shanghai, Beijing). The pH of the rumen fluid was 6.2 ± 0.21. It took 20 min to return to the laboratory from the dairy farm.

### 2.2. Experimental Design and In Vitro Batch Culture

In vitro experiment was conducted, as described previously [26] by glass bottles (volume capacity, 120 mL), combined with a rumen simulation system for gas production recording (AGRSIII, Beijing, China). The fermentation system included 1 g of substrate, 50 mL of buffer solution, and 25 mL of rumen fluid. The substrate was the TMR fed to the donors of rumen fluid and dried at 65 °C for 48 h in a forced-air oven, ground in a Wiley mill (Thomas–Wiley model 4 Wiley mill, Norcross, Thorofare, NJ, USA) to pass a 2 mm sieve. The buffer solution was formulated with 15.7 mg/L CaCl_2_•2H_2_O, 11.89 mg/L MnCl_2_•4H_2_O, 1.19 mg/L CoCl_2_•6H_2_O, 9.51 mg/L FeCl_2_•6H_2_O, 8.32 g/L NaHCO_3_, 0.95 g/L NH_4_HCO_3_, 1.36 g/L Na_2_HPO_4_, 1.47 g/L KH_2_PO_4_, 0.14 g/L MgSO_4_•7H_2_O, 1.19 mg/L resazurin, and 0.30 g/L Na_2_S•9H_2_O (Sinopharm Chemical Reagent Co., Ltd., Shanghai, China). The rumen fluid was a mixture of an equal volume of rumen fluid from three dairy cows. The value of rumen degraded protein (RDP) in the substrate and RDP requirement of microbiota were estimated based on the NRC [29]. The RDP in the substrate and RDP requirement of microbiota was 9.90% and 10.4%.

Treatments included the control (no additive, CON), 5 mg urea (UR), 25.08 mg Met, and 23.57 mg MD. The urea addition in the UR group was determined to meet the RDP requirement, and the Met addition and the MD addition were calculated to match the nitrogen supplementation from urea. The urea product was purchased from KELUNDUO Food Agricultural Co., Ltd. (Lianyungang, China) with a purity of 99%. The Met product was purchased from Bluestar Adisseo Co., Ltd. (Nanjing, China) with a purity of 99%. MD, with a purity of 99%, was purchased from Hebei Tianma-Muge Biotechnology Co., Ltd. (Hengshui, China). The 48 bottles with six replicates per time (24 h, 48 h) per treatment (UR, Met, MD, CON) were immediately sealed after introducing anaerobic N_2_ for 5 s, and then connected to the recording system at 39 ℃ to reduce the variability [27].

After 48 h and 24 h fermentation, the solid fraction in each bottle was collected by nylon bag (80 mm × 150 mm size, 42 μm pores), and the residual culture fluid was collected into 2.5 mL microtubes and 15 mL centrifuge tubes for microbial composition and fermentation parameters analyses. The solid content was then dried in a nylon bag as mentioned above and prepared for nutrient composition analyses. The pH of the filtered culture fluid was measured after filtration.

The nutrient compositions of original TMR and residual solid fraction were determined according to a previously described method [30] including dry matter (DM), crude protein (CP), neutral detergent fiber (NDF), and acid detergent fiber (ADF).

The culture fluid for volatile fatty acid (VFA) and ammonia nitrogen (NH_3_-N) concentrations were measured by gas chromatography (Agilent 6890N, Agilent Technologies, Inc., Beijing, China) and Multiskan SkyHigh microplate reader (Thermo Fisher Scientific, Shanghai, China) according to Kong et al. [31].

### 2.3. DNA Extraction and Sequencing

Bacterial DNA in the culture fluid was extracted using an Omega Stool DNA kit (Omega Bio-Tek, Norcross, GA, USA) according to the instruction. One of the microtube was damaged due to contraction in the liquid nitrogen, five replicates were eventually used for the bacterial composition analysis. Both quality and quantity of DNA were evaluated using a NanoDrop 2000 spectrophotometer (NanaDrop Technologies, Wilmington, DE, USA). Amplicon library preparation was performed by polymerase chain reaction (PCR) of the V3-V4 region of the 16S rRNA gene using the universal primers 338F (5′-ACTCCTACGGGAGGCAGCAG-3′), and 806R (5′-GGACTACHVGGGTWTCTAAT-3′) [32]. The reaction system included 10 ng of template DNA, 4 μL of FasPfu buffer (CoWin Biosciences, Inc., Cambridge, MA, USA), 2 μL of 2.5 mmol/L dNTPs, 0.8 μL of each primer, 0.4 μL of FasPfu polymerase (CoWin Biosciences, Inc., Cambridge, MA, USA), 0.2 μL of bovine serum albumin, and double-distilled H_2_O to make up the volume to 20 μL. The expected product length was about 440 bp. Amplicons were electrophoresed as the description above, purified using an Agencourt AM Pure XP kit (Beckman Coulter Life Sciences, Indianapolis, IN, USA), and quantified using the QuantiFluor^TM^-ST system (Promega, Madison, WI, USA), and paired-end sequenced on an Illumina MiSeq platform PE250 (Illumina, Inc., San Diego, CA, USA). The sequencing data were deposited in the NCBI Sequence Read Archive (SRA) under accession numbers from SAMN19128494 to SAMN19128513 in PRJNA699978.

The Microbial Ecology (QIIME) program software was used to process raw data [33]. Briefly, paired-end forward and reverse reads were joined, and then primers and homopolymer runs (maximum length, 8) of sequences were trimmed. Only sequences ≥ 400 bp in length, with average quality score ≥ 25 and with ambiguous bases ≤ 6 remained for downstream analysis. Ne Novo chimera checking was performed using UCHIME (Tiburon, CA, USA) [34]. After quality control, the sequences were assigned to operational taxonomic units (OTUs) at a 97%-identity threshold using UPARSE (version 10.0.240, Tiburon, CA, USA) [35]. Sequences were assigned against the SILVA 138 bacterial alignment database (Bremen, Germany) using the Ribosomal database project classifier with a confidence threshold of 70% [36].

### 2.4. Statistical Analysis

The gas production data were used to calculate kinetics parameters of gas production by NLIN procedure in SAS 9.2 (SAS Institute Inc., Cary, NC, SAS) according to an exponential model, as follows:

(1) $${\mathrm{GP}=\mathrm{Max}\mathrm{GP}\times [1-e}^{-\mathrm{Frac}\mathrm{GP}\times \left(\mathrm{time}-\mathrm{Lag}\right)}]$$

where GP (mL) is gas production, Max GP (mL) is the ideal maximum gas production, Frac GP (h^−1^) is the gas production rate, and Lag (mL) is the lag phase before gas production commences [37]. The time to reach half of the ideal maximum gas production (HT, h), and the average gas production rate when half of the ideal maximum gas (AGPR, mL/h) were calculated using the Max GP, Frac GP, and Lag values [38]:

(2) $$\mathrm{HT}=\log\left(\frac{2}{\mathrm{Frac}\mathrm{GP}}\right)+\mathrm{Lag}$$

(3) $$\mathrm{AGPR}=\frac{\mathrm{Max}\mathrm{GP}\times \mathrm{Frac}\mathrm{GP}}{2\times (\log\left(2\right)+\mathrm{Frac}\mathrm{GP}\times \mathrm{Lag})}$$

Fermentation kinetics parameters, nutrient digestibility, pH value, NH_3_-N concentration, and VFA data were analyzed using the GLM procedure in the SAS v9.2 software. Nutrient digestibility at different times was analyzed using the GLM produce to obtain *p*-value of treatment, time, and the interaction effect of time and treatment using the following model:

(4) $$Y_{ijk}=\mu +P_{i}+T_{j}+T_{j}\times P_{i}+B_{k}+\varepsilon_{ijk}$$

where *Y_ijk_* is the dependent variable; *μ* is the overall mean; *P_i_* is the fixed effect of diet (CON, MD, Met, UR); *T_j_* is the fixed effect of time (24 h, 48 h); *T_j_* × *P_i_* is the interaction between *T_j_* and *P_i_*; *B_k_* is the random bottle effect, and *ε_ijk_* is the model error.

The fermentation kinetics parameters, pH value, NH_3_-N concentration, and VFA data were analyzed to obtain the *p*-value of the treatments. Main effects and interactions were declared significant at *p* < 0.05, and trends were declared at 0.05 ≤ *p*-values ≤ 0.10.

### 2.5. Sequencing Data Analysis

The OTU table and Taxonomy table were uploaded on the Microbiome Analyst platform [39] (released in February 2021 and available at https://www.microbiomeanalyst.ca (accessed on 4 May 2021)). Low count filter and low variance filter were conducted to remove low-quality or chimeric features. The OTUs including 20% of read counts greater than four counts were retained, and the variance of read counts was measured using the inter-quantile range, and the lowest percentage based on the cutoff (>10%) was excluded. All samples were rarefied to even sequencing depth based on the sample which had the lowest sequencing depth. Differences in α diversity indices (Chao1, OTU number, Simpson, Channon indices) were analyzed with the Kruskal–Wallis test on the Microbiome Analyst platform [39].

Principal coordinates analysis (PCoA) combined with non-parametric multivariate variance (PERMANOVA) was analyzed based on the Bray–Curtis distance matrices. The heatmap clustering plot and stacked bar chart were generated at the phylum level using the Microbiome Analyst [39]. The linear discriminant analysis (LDA) effect size (LEfSe) tool was used to detect features with significant differential abundance using the non-parametric Kruskal–Wallis sum-rank test and effect size on the Microbiome Analyst platform [39]. A cut-off value ≥ 2 and <0.05 was used for linear discriminant analysis (LDA) score and *p*-value, respectively.

Spearman’s correlation test was used to assess the correlation between fermentation parameters and the selected microbial genera. The analysis was performed by SPSS software (version 20.0; IBM, Armonk, NY, USA), and was plotted using GraphPad Prism 7 (GraphPad Software, San Diego, CA, USA). The selected genera were obtained from LEfSe analysis with enrichment in UR, Met, and MD groups.

## 3. Results

### 3.1. Nutrient Digestibility

**Figure 1:**
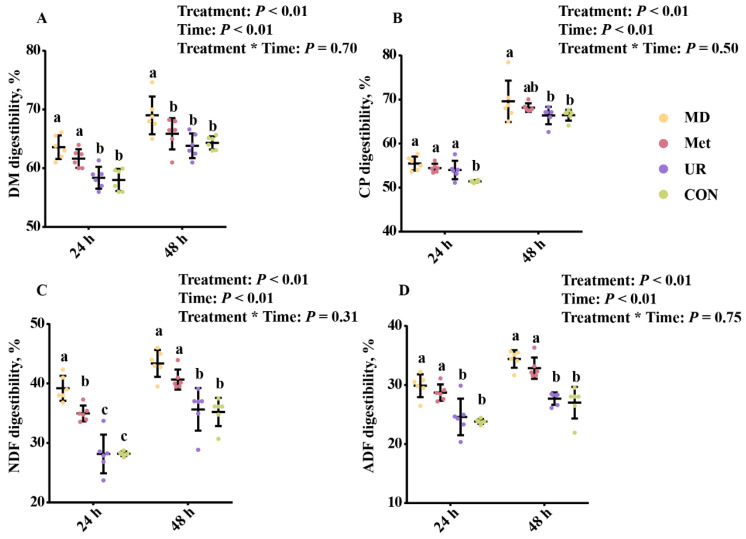
Effects of urea, methionine, and methionine dipeptide on in vitro digestibility of dry matter (**A**), crude protein (**B**), neutral detergent fiber (**C**), and acid detergent fiber (**D**) of total mixed ration at 24 h and 48 h. CON = no additive, UR = urea addition, Met = methionine addition, and MD = methionine dipeptide addition. Means with different letters (a–c) at a given time are significantly different (*p* < 0.05). Vertical bars denote standard deviation (SD). DM, dry matter; CP, crude protein; NDF, neutral detergent fiber; ADF, acid detergent fiber; *n* = 6 per treatment per time.

The nutrient digestibility results are shown in Figure 1. There were no significant differences between the UR and CON groups at 48 h (Figure 1; *p* > 0.10). Both DM digestibility and CP digestibility at 24 h were higher in the Met group than in the CON group (Figure 1A,B; *p* < 0.05), and no difference was observed at 48 h (Figure 1A,B; *p* > 0.10). DM and CP digestibility at 48 h were higher in the MD group than in the UR and CON groups (Figure 1A,B; *p* < 0.05). NDF and ADF digestibility at 24 h and 48 h were higher in the MD and Met groups than in the UR and CON groups (Figure 1C,D; *p* < 0.05). There was no interaction effect on nutrient digestibility (Figure 1; *p* > 0.10).

### 3.2. Fermentation Kinetics Parameters

**Figure 2:**
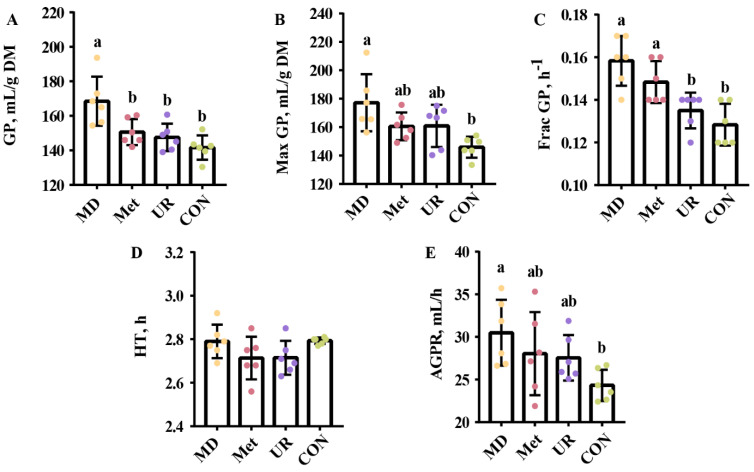
Effects of urea, methionine, and methionine dipeptide on parameters of in vitro fermentation kinetics of total mixed ration during 48 h incubation. CON = no additive, UR = urea addition, Met = methionine addition, and MD = methionine dipeptide addition. GP (**A**) indicates cumulative gas production at 48 h. Max GP (**B**) indicates ideal maximum gas production. Frac GP. (**C**) indicates the fractional rate of gas production. HT (**D**) indicates time to reach half the ideal maximum gas production. AGPR (**E**) indicates the average gas production rate when half of the ideal maximum gas production was produced. Means with different letters (a, b) are significantly different (*p* < 0.05). Vertical bars denote standard deviation (SD); *n* = 6 per treatment per time.

Figure 2 shows the effects of urea, Met, and MD on fermentation kinetics parameters. The parameters in the CON and UR groups were not different (*p* > 0.10). Gas production in the MD group was higher than in the rest (Figure 2A; *p* < 0.05). There was no significant difference in Max GP, Frac GP, and AGPR between the MD and Met groups (Figure 2B,C,E; *p* > 0.10). However, Max GP, Frac GP, and AGPR in the MD group were higher than in the CON group (Figure 2B,C,E; *p* < 0.05).

### 3.3. pH Value, NH3-N Concentration, and VFA Proportion

**Figure 3:**
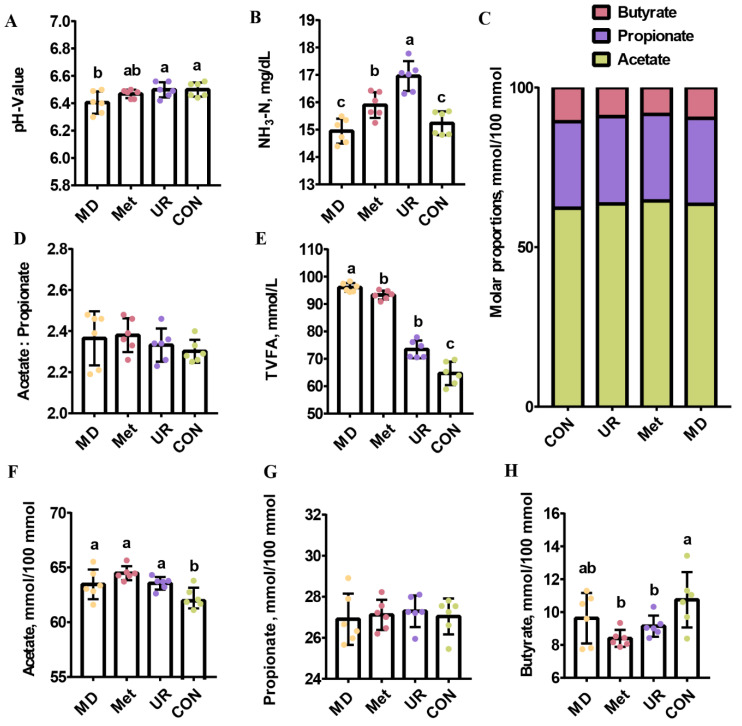
Effects of urea, methionine, and methionine dipeptide on in vitro pH value (**A**), ammonia concentration (**B**), and volatile fatty acid composition (**C**–**H**) of total mixed ration during 48 h incubation. CON = no additive, UR = urea addition, Met = methionine addition, and MD = methionine dipeptide addition. Individual volatile fatty acids are represented as molar proportions (mmol/100 mmol. **F**–**H**). Means with different letters (a–c) are significantly different (*p* < 0.05). Vertical bars denote standard deviation (SD); *n* = 6 per treatment per time.

Figure 3 shows an overview of the effects of urea, Met, and MD on fermentation parameters. The pH value in the MD group was lower than in the UR and CON groups (Figure 3A; *p* < 0.05). The NH_3_-N concentration was higher in the UR group than in the other groups (Figure 3B; *p* < 0.05), and was higher in the Met group than in the MD and CON groups (Figure 3B; *p* < 0.05).

There was no difference between these groups in terms of molar proportion of propionate and the ratio of acetate to propionate (Figure 3D,G; *p* > 0.10). Total volatile fatty acid (TVFA) in the MD group was higher than in the other groups (Figure 3E; *p* < 0.05), and that in the Met and UR groups were higher than in the CON group (Figure 3E; *p* < 0.05). The molar proportion of acetate was lower in the CON group than in the other groups (Figure 3F; *p* < 0.05). The molar proportion of butyrate was not affected by MD treatment (Figure 3H; *p* > 0.10), while it was lower in the Met and UR groups than in the CON group (Figure 3H; *p* < 0.05).

### 3.4. Diversity of Microbiota

**Figure 4:**
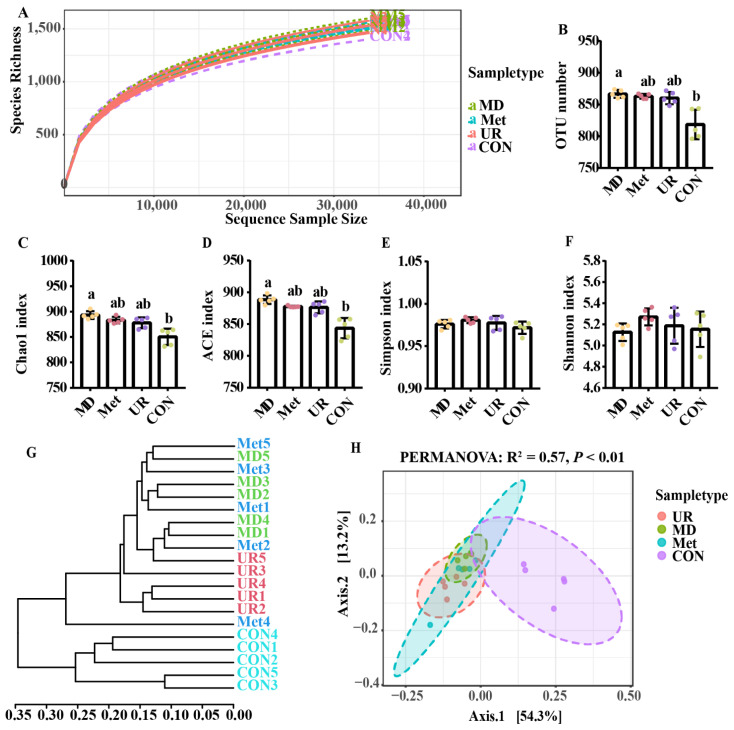
Rarefaction curves (**A**), OTU number (**B**), α diversity (**C**–**F**), dendrogram analysis (**G**), and β diversity (**H**) in response to the microbiota with urea, methionine or methionine dipeptide in vitro, after 48 h fermentation. CON = no additive, UR = urea addition, Met = methionine addition, and MD = methionine dipeptide addition. All analyses were based on the OTU level. A principal coordinate analysis plot based on the Bray–Curtis distances was conducted. *p*-value obtained using non-parametric multivariate of variance (PERMANOVA) based on Bray–Curtis distances (**H**). Means with different letters are significantly different (*p* < 0.05). Vertical bars denote standard deviation (SD); *n* = 5 per treatment per time.

After removing low-quality reads, 2,282,354 clean reads were subjected to subsequent analysis. The rarefaction curves showed the current sequencing depth to be sufficiently representative of the microbiota (Figure 4A). When α-diversity indices were compared, no difference was observed in the richness of the microbiota across the CON, UR, and Met groups (Figure 4B–D, *p* > 0.10), while the richness of the microbiota in the MD group was higher than in the CON group (Figure 4B–D, *p* < 0.05). There was no difference in evenness across the treatments (Figure 4E,F; *p* > 0.05). A dendrogram analysis (Figure 4G) and PCoA plot (Figure 4H) based on the Bray–Curtis distances showed distinct clustering between the CON group and others (PREMANOVA: *p* < 0.01); there was a close connection between the UR, Met, and MD groups (Figure 4G,H).

### 3.5. Microbial Composition and Its Comparison in Response to Urea, Methionine, and Methionine Dipeptide

**Figure 5:**
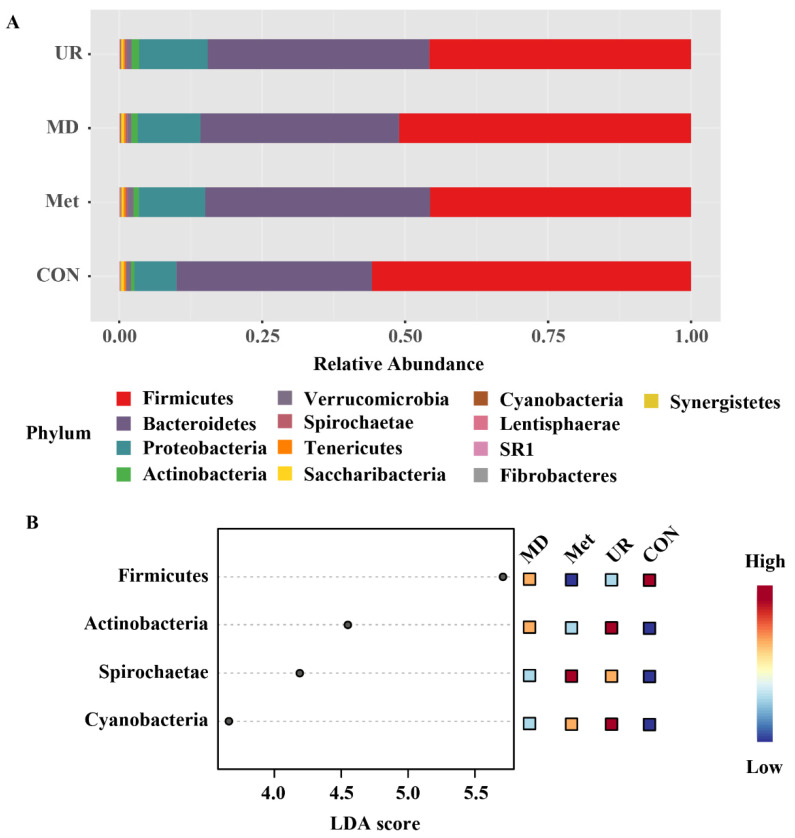
Phylum composition (**A**) of the microbiota and linear discriminant analysis effect size (LEfSe) (**B**) identifying differential phyla of microbiota in response to urea, methionine, or methionine dipeptide supplementation. CON = no additive, UR = urea addition, Met = methionine addition, and MD = methionine dipeptide addition. A cut-off value ≥ 2 was used for the linear discriminant analysis (LDA) score. *n* = 5 per treatment per time.

The phylum compositions and comparisons are shown in Figure 5. Among the phyla, Firmicutes (49.56%), Bacteroidetes (36.78%), Proteobacteria (10.44%), and Actinobacteria (1.00%) were predominant; the remaining minor phyla (relative abundance < 1%) are presented in Figure 5A. To identify the specific bacterial phyla associated with Met and MD, we conducted the LEfSe analysis (Figure 5B). Firmicutes was enriched in the CON group (*p* = 0.02, LDA score = 5.71), Actinobacteria and Cyanobacteria were enriched in the UR group (Actinobacteria: *p* = 0.03, LDA score = 4.55; Cyanobacteria: *p* = 0.01, LDA score = 3.66), and Spirochaetae was enriched in the Met group (*p* = 0.01, LDA score = 4.19) (Figure 5B).

**Figure 6:**
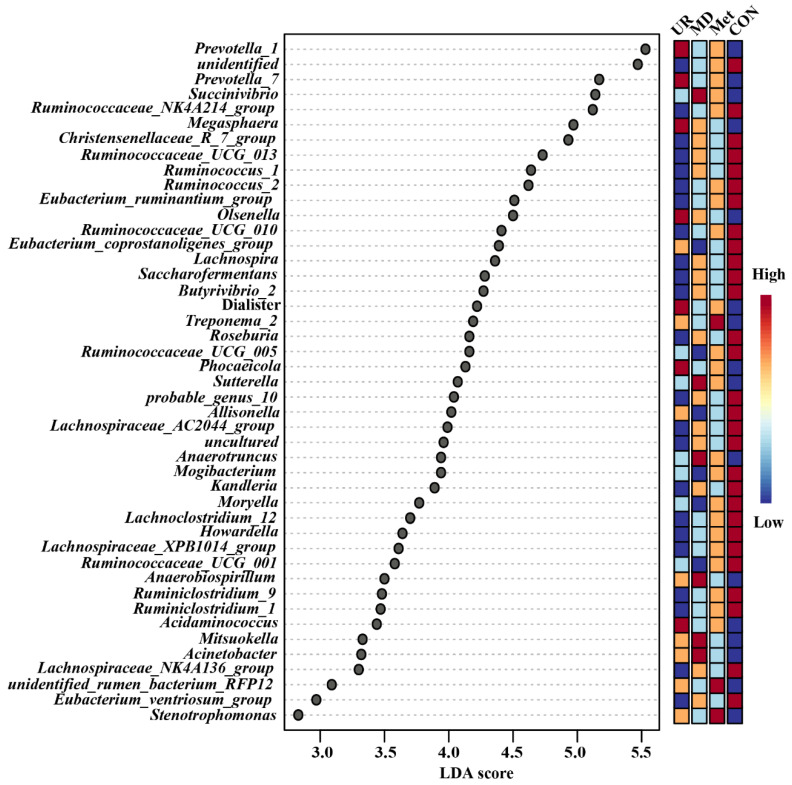
Linear discriminant analysis effect size approach identifying most differential genera in microbiota in response to urea, methionine, and methionine dipeptide supplementation. CON = no additive, UR = urea addition, Met = methionine addition, and MD = methionine dipeptide addition. A cut-off value ≥ 2 was used for the linear discriminant analysis (LDA) score. Only the top 45 significantly different genera are shown; *n* = 5 per treatment per time.

LEfSe analysis was used to select the significantly different genera. Overall, 29 of the top 45 genera were significantly enriched in the CON group (Figure 6; *p* < 0.05) and only 3 and 6 genera were enriched in the MD and Met groups, respectively (Figure 6).

**Figure 7:**
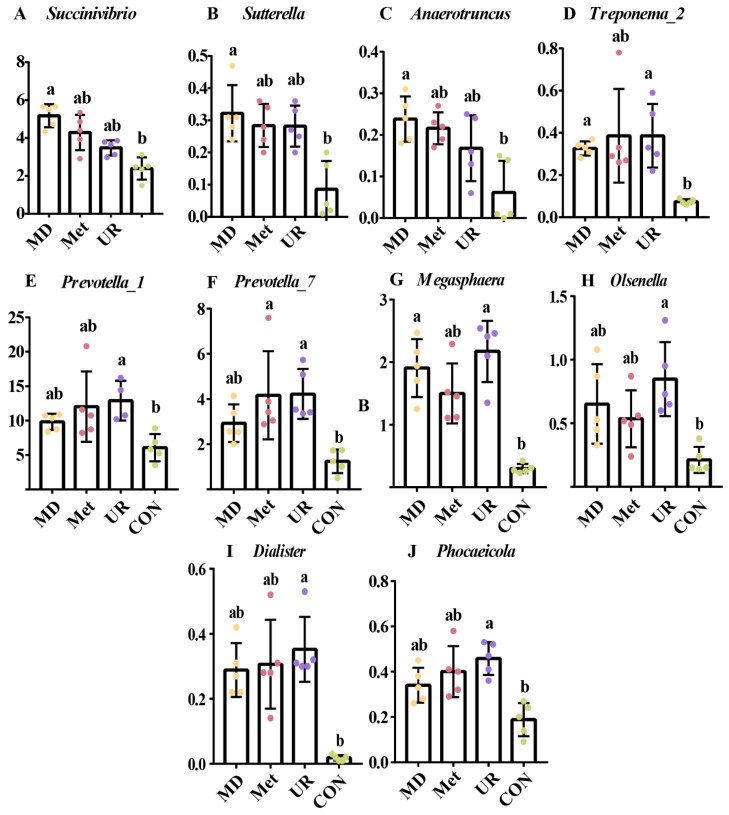
The significantly different genera response to methionine dipeptide (**A**–**C**), methionine (**D**), and urea (**E**–**J**) supplementation. CON = no additive, UR = urea addition, Met = methionine addition, and MD = methionine dipeptide addition. These genera were tested by linear discriminant analysis effect size and the relative abundance analyzed in detail by the Kruskal–Wallis test. Means with different letters (a, b) are significantly different (*p* < 0.05). Vertical bars denote standard deviation (SD); *n* = 5 per treatment per time.

Subsequently, we summarized the relative abundance of the genera in detail, considering those that were enriched in the UR, Met, and MD groups (Figure 7). Relative abundances of *Succinivibrio*, *Sutterella*, and *Auaerotruncus* were higher in the MD group than in the CON group (Figure 7A–C; *p* < 0.05). There was no difference in the Relative abundance of *Treponema_2* among the UR, Met, and MD groups (Figure 7D; *p* > 0.10), while *Treponema_2* was enriched in the Met group and higher than in the CON group (Figure 7D; *p* < 0.05). No significant differences in the relative abundance of *Prevotella_1*, *Prevotella_7*, *Megasphaera*, *Olsenella*, *Dialister*, and *Phocaeicola* were found among UR, Met, and MD groups (Figure 7E–J; *p* > 0.10), whereas these were higher in the UR group than in the CON group (Figure 7E–J; *p* < 0.05).

### 3.6. Correlation Analysis between Microbial Features and Nutrient Digestibility, Gas Production Parameters, and Fermentation Parameters

**Figure 8:**
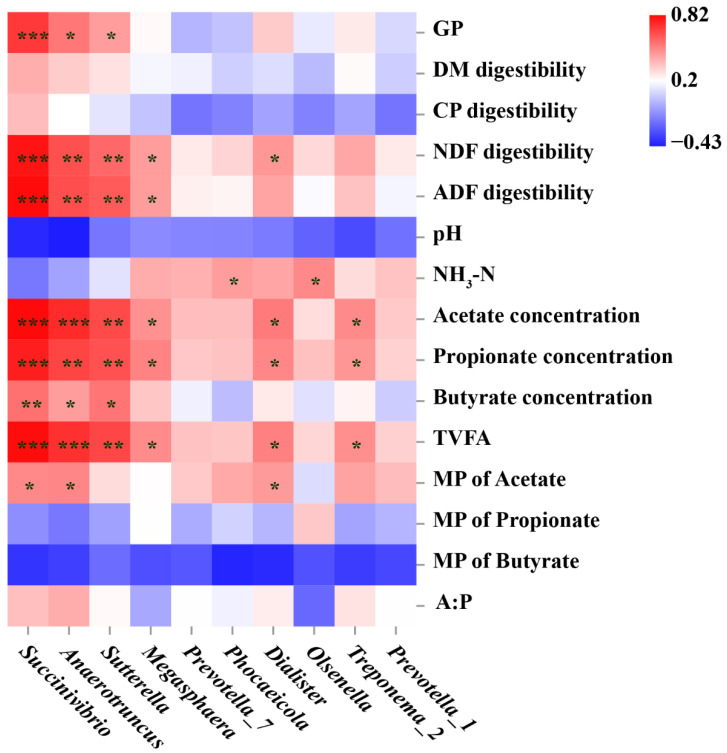
Spearman’s correlation between the relative abundance of genera and phenotypic indices. Genus were obtained using linear discriminant analysis effect size and found to be enriched in UR, Met, and MD groups. CON = no additive, UR = urea addition, Met = methionine addition, and MD = methionine dipeptide addition. Colors represent the correlation coefficient. Red represents a positive correlation, and blue represents a negative correlation. A dark color represents a stronger correlation, whereas a light color represents a weaker correlation. *, 0.01 < *p* < 0.05; **, 0.001 < *p* < 0.01; ***, *p* ≤ 0.001; GP, gas production; DM, dry matter; CP, crude protein; NDF, neutral detergent fiber; ADF, acid detergent fiber; TVFA, total volatile fatty acid; MP, molar proportion; A:P, acetate concentration: propionate concentration.

The correlation between the relative abundance of features and phenotypic indices is shown in Figure 8. The relative abundance of *Succinivibrio*, *Anaerotruncus*, and *Sutterella* was positively correlated with gas production, NDF, and ADF digestibility, acetate, propionate, butyrate, and TVFA concentration (*p* < 0.05). Furthermore, the molar proportion of acetate was positively correlated with *Succinivibrio*, *Anaerotruncus*, and *Dialister* (*p* < 0.05). The concentrations of acetate, propionate and TVFA were positively correlated with the relative abundance of *Megasphaera*, *Dialister*, and *Treponema_2* (*p* < 0.05).

## 4. Discussion

Rumen fermentation is important for dairy cows to utilize the nutrition from otherwise-indigestible plant polymers and compounds [40]. Several studies have indicated that the rumen-protected amino acids and peptides could enhance rumen fermentation ability and alter the rumen microbiota profile [13,20,21,22,41]. The present study aimed to explore the effects of MD on rumen fermentation and bacterial composition, and further investigated the potential of MD in performance improvement. In vitro fermentation technique was used in this study and it allows for the setting of experimental conditions more diverse and precise than in vivo experiments. However, the limiting of in vitro fermentation and in vivo studies are needed to consider and further investigate.

One of the important findings of the current study was the different responses in dry matter digestibility and gas production to different nitrogen-containing compounds. The TMR ruminal degradation rate was enhanced by the supplement with Met and MD, especially MD had the best positive effects on TMR decomposition in the rumen. However, urea had no promoting effects on TMR degradation. Distinct nitrogen forms have different functions during the rumen fermentation process [42]. An in vitro study indicated that adding urea higher than 50 mg/L can increase microbial protein yield under nitrogen-limiting conditions [43]. Further analysis found that amino acid mixtures and peptides stimulated microbial growth, and resulted in higher microbial growth than urea [20]. Several studies focused on evaluating the effects of rumen-protected Met on nutrient digestibility and found NDF digestibility [16], and DM digestibility [44] of TMR, and organic matter digestibility of feed ingredients [45] were enhanced. The Met results are consistent with those of our study. In contrast, few studies have tested the effects of MD on rumen fermentation and dairy nutrition. Our in vitro results indicate that MD is more effective in enhancing fiber digestion and nitrogen digestion and hence may have the potential to improve milk production. Therefore, in vivo feeding experiments are needed to further test the apparent digestibility of nutrients. Note that the effects of MD on different stages of dairy cows may be different from our study as the rumen fluid used in our study was obtained from mid-lactating dairy cows.

The high fermentability of fiber produces a large amount of VFA to reduce the pH value, which is consistent with our results of nutrient digestibility and gas production. An inconsistent result was obtained from NH_3_-N concentration. Abbasi et al. [16] found that CP digestibility, microbial protein concentration, and NH_3_-N concentration increased with both low and high Met supplementation. However, Baghbanzadeh-Nobari et al. [45] showed that Met supplementation decreased NH_3_-N concentration and increased CP digestibility. These results may due to the different conversion rates of NH_3_-N to microbial protein based on different Met-dose or basic diets. Dietary protein, urea, and other nitrogen-containing compounds are degraded to peptides and amino acids and eventually deaminated into NH_3_-N or incorporated into microbial proteins [42]. Hence, the NH_3_-N concentration depends on the balance between the consumption of microbial proteins and the yield from nitrogen-containing compounds. In this regard, we speculate that the MD supplementation may facilitate microbial protein synthesis from degraded protein, which is the precursor of body protein and milk protein [7].

Microorganisms in the rumen are intermediaries between the dietary treatment and the substrate. Hence, pH, NH_3_-N, and VFA changes can be attributed to the variation in microbiota. Our data showed Firmicutes, Bacteroidetes, Proteobacteria, and Actinobacteria, to be prevalent regardless of the treatment. These results were consistent with previous results [31]. Our β-diversity, dendrogram analysis, and phylum composition visualized by heatmap plot further showed the UR, MD, and Met groups were clustered together, separated from the CON group. The significant effect of different nitrogen sources on bacterial composition has also been reported. The addition of extra nitrogen sources to the fermenter or the diet, such as urea [43,46,47], Met [48], and lysine [41] stimulate microbial growth and change microbiota. Our valuable finding was obtained from α-diversity, which suggests that extra nitrogen sources possibly stimulate a portion of bacteria instead of all the bacteria cumulatively. Metagenomic analysis observed that urea addition in sheep diet significantly increased the relative abundance of genera involved in nitrogen metabolism especially. The bacterial composition was also altered by lysine supplementation to support energy metabolism, in which the microbial diversity was unchanged [41]. Therefore, we speculate that the supplementation of urea, Met, and MD may facilitate the growth of specific bacteria (microbial richness) in different ways.

In the current study, higher TVFA concentrations after different addition were observed. Li et al. [46] used urea to formulate different levels of CP in the diets of Hu sheep and showed the genera, *Prevotella* and *Megasphaera*, to be enriched, whereas *Ruminococcus* and *Butyrivibrio* were enriched in the no-urea group, which were consistent with our findings. The predominant genus, *Prevotella* in the rumen utilizes a wide range of substrates, such as starch, hemicellulose, proteins, peptides, and amino acids [49,50]. Notably, *Prevotella* is an important microbial member involved in the degradation of peptides into amino acids, which is regarded as the limiting process in proteolysis [50,51] and maybe attributed to increased CP digestibility in our study.

Other genera, *Megasphaera* and *Olsenella* with high relative abundance, showed a 3- to 7-fold change after urea, Met, and MD supplementation. Interestingly, lactate is the dominant product from *Olsenella*, through the action of β-glucosidase [52]. A classic isolate of the genus, *Megasphaera*, can convert lactate to butyrate [53]. Theoretically, butyrate concentration should be increased after MD and Met addition. However, the genus *Butyrivibrio* was enriched in the CON group and can grow on a range of carbohydrates with butyrate production, such as starch and hemicelluloses [54]. The different butyrate-producing bacteria may lead to the invariable butyrate proportion among four groups and another possible reason is that butyrate accounts for a relatively smaller proportion of TVFA, and may be masked by the modification of acetate and propionate. Furthermore, Pitta et al. found that the relative abundance of *Megasphaera* was decreased by Met analog supplementation after 28 days in dairy cows when exposed to diets with risk for milk fat depression. We speculate that the relatively shorter time (48 h) used in our study was limited and further long term should consider further.

Interestingly, the relative abundance of *Ruminococcaceae_NKA214_group*, *Ruminococcaceae_UCG_013*, *Ruminococcaceae_1*, *Ruminococcaceae_2*, and *Ruminococcaceae_010* in the UR group were the lowest among all groups. The ruminal bacteria, *Ruminococcus* spp., are capable of degrading cellulose [55,56], hence suggesting that the relative abundance of *Ruminococcus* may be associated with NDF and ADF digestibility. In our study, the NDF and ADF digestibility by urea treatment were decreased than Met and MD treatment. Li et al. [46] observed a decrease in the relative abundance of *Ruminococcus* in the microbiota of a non-urea group. Although some of the genera *Ruminococcaceae* were enriched in CON group, the lowest out number in the CON group indicates the actual amount of bacteria, including *Ruminococcus*, may be far from that in the other groups. Hence, we speculate that *Ruminococcus* may not be sensitive to urea addition.

Higher relative abundance of *Anaerotruncus* and *Treponema,* two potential cellulolytic bacteria [57,58,59], were observed in the MD group. In addition to the above genera, the relative abundance of *Succinivibrio* in the rumen has been reported to be positively associated with feed efficiency in beef cattle [60], and with milk production [61] and milk protein yield [62] in dairy cows due to its ability of produce succinate, the precursor of glucose. Although our results showed that MD contributed to a higher relative abundance of *Succinivibrio* in in vitro study, further animal studies are required to confirm whether MD plays a role in milk production or feed efficiency.

## 5. Conclusions

In conclusion, the present study used 16S rRNA gene analysis and a gas recording system to examine the microbial composition and fermentation characteristics under a TMR with different nitrogen-containing sources. A comparison of nutrient digestibility showed that MD, followed by Met, was most effective in enhancing TMR utilization, especially in respect of dry matter digestibility. The stimulatory effect of MD on bacterial growth efficiency was emphasized by the high richness. Our results showed that extra nitrogen supplementation increased the relative abundance of *Prevotella*, *Megasphaera*, and *Olsenella*, whereas MD enriched *Succinivibrio* and *Anaerotruncus*. The results highlight the possible role of MD in improving nutrient utilization in the rumen and provide a basis of MD on dairy performance. Multiple factors (e.g., dose, lactation period) taken into consideration are essential to the use of MD.

## Figures and Tables

**Table 1:** The ingredients and nutrient composition of total mixed ration fed to cows and the substrate, % of dry matter basis.

Items	Contents
Ingredients	
Alfalfa	9.34
Alfalfa silage	2.12
Corn silage	25.21
Steam-flaked corn	17.82
Corn	12.73
Fatty powder	1.27
Soybean meal	15.28
Soybean hull	4.24
Corn gluten meal	2.55
Cottonseed meal	6.37
NaHCO_3_	0.64
Molasses	1.05
3% premix ^1^	1.38
Total	100
Nutrient level ^2^	
DM, % of air-dried weight	94.55 ± 0.37
CP	16.00 ± 0.35
NDF	38.18 ±0.91
ADF	25.32 ± 0.43

^1^ One kg premix contained the following: VA, 130,000 IU; VE, 465 IU; Cu, 2600 mg; Mn, 6000 mg; Zn, 2600 mg; Se, 70 mg; I, 120 mg; Co, 70 mg. ^2^ All data were obtained from chemical analysis and shown as mean ± standard deviation. DM, dry matter; CP, crude protein; NDF, neutral detergent fiber; ADF, acid detergent fiber

## Data Availability

The sequencing data we generated were deposited in the NCBI SRA under accession numbers from SAMN19128494 to SAMN19128513 in PRJNA699978.
